# Safety and Feasibility of Same-Day Discharge After Primary Bariatric Surgery and the Value of Remote Monitoring with the Healthdot

**DOI:** 10.1007/s11695-025-07828-2

**Published:** 2025-03-27

**Authors:** Kayleigh Ann Martina van Dam, Geert Henricus Jozef Martinus Verkoulen, Pieter Petrus Henricus Lucien Broos, Evelien de Witte, Jan Willem M. Greve, Evert-Jan Gijsbert Boerma

**Affiliations:** 1 Zuyderland Medisch Centrum, Sittard, Netherlands; 2 Maastricht University, Maastricht, Netherlands; 3 Nederlandse Obesitas Kliniek, Zeist, Netherlands

**Keywords:** Same day discharge, Healthdot, Remote monitoring

## Abstract

**Background:**

Annually, more than 12,500 bariatric procedures are performed in the Netherlands. Same-day discharge (SDD) has been implemented in several surgical procedures and was recently introduced in bariatric metabolic surgery (BMS). However, the best way to safely facilitate this introduction is a subject of debate. This study aims to assess the feasibility of SDD in selected patients undergoing primary BMS and the value of remote monitoring with a wireless vital sign monitoring system (Healthdot).

**Methods:**

This retrospective study included all primary sleeve gastrectomy and Roux-en-Y gastric bypass procedures in the first year after the introduction of SDD in a large teaching hospital in the Netherlands. SDD patients were remotely monitored postoperatively for 7 days using the Healthdot which measured vital signs continuously. The primary outcome was the success rate of SDD. Secondary outcomes included readmissions, complication rate, and Healthdot alarms.

**Results:**

Out of 813 consecutive primary bariatric procedures between March 2022 and March 2023, there were 514 eligible patients of whom 260 were scheduled for SDD. Successful SDD was achieved in 246 patients (94.6%). Eight patients were readmitted within 48 h resulting in a success rate of 91.5%. Among 217 patients (83.5%) who went home with the Healthdot device, 28 alarms occurred of which 26 did not have a complication. In 11 other patients, a complication occurred without any alarms during the 7-day period.

**Conclusions:**

SDD after primary bariatric procedures is considered safe if specific selection and discharge criteria are maintained. The Healthdot is found to be ineffective in predicting complications in this patient group.

## Introduction

As a consequence of the rising worldwide prevalence of obesity, there has been an increase in the popularity of anti-obesity treatment, among which surgical procedures [1]. Between 2015 and 2022, 103,336 bariatric metabolic procedures have been performed in the Netherlands of which 74.1% were classified as primary procedures [2]. In bariatric metabolic surgery (BMS), the perioperative process has been improved through the implementation of fast-track surgery (ERABS), which reduces the length of hospital admission [3–5]. The fast-track protocol has also proven to be safe in conversional BMS [6]. To meet the increasing demand for BMS in the light of decreasing health care capacity, the potential of same-day discharge (SDD) is being studied, especially for primary procedures [7].

Medical devices for remote monitoring are considered an innovation to facilitate the safe introduction of SDD. The value of these devices has already been studied in combination with orthopedic and general surgery [8, 9]. However, the use of remote monitoring in SDD for BMS has been limited to a few studies and in various forms as there is no consensus on the optimal method [10–12]. One of the possible methods is a wearable remote monitoring device called the Healthdot, which is developed by Philips. The Healthdot is a single-use wearable biosensor which is affixed to the skin just below the left side of the ribcage. It continuously measures the patient’s heart rate and respiratory rate and offers real-time monitoring through a clinical information system. The Healthdot has been studied in abdominal cancer surgery where it was found to be comparable to the Modified Early Warning Score (MEWS) measured in the hospital [13]. Its use in bariatric patients has not been previously published, although a clinical trial in the Netherlands is still undergoing [14].

The aim of this study was to assess the safety and effectiveness of SDD and remote monitoring with the Healthdot device in the first year after introduction in a large teaching hospital.

## Methods

### Study Design

Data was retrospectively collected from all consecutive patients who underwent a primary bariatric procedure, either a sleeve gastrectomy (SG) or a Roux-en-Y gastric bypass (RYGB), in a 1-year period between March 2022 and March 2023.

### Patient Selection and Perioperative Protocol

Patients were included if they underwent either a primary RYGB or SG procedure between March 2022 and March 2023 at Zuyderland Medical Center. Prior to surgery, the patients were screened for eligibility for SDD following the criteria presented in Table 1. These criteria were used to exclude patients with a potentially higher risk for complications. Not all patients eligible for SDD were treated as such due to capacity issues.

**Table 1:** Preoperative selection criteria for eligibility of same-day discharge and remote monitoring with the Healthdot

Inclusion	Exclusion
-BMI < 50 kg/m^2^ -Age ≥ 18 years -Primary procedure (no revisional/conversional surgery) -Residing within a maximum of 30-min travel time to the hospital -** ≥ **3.5 kg weight loss compared to screening weight -Availability of a (Dutch speaking) caregiver for the first 24 h following discharge -Patients can be reached by phone during the 7-day monitoring period	**-**Patients not residing in the Netherlands* **-**History of endocrinologic, cardiovascular, and/or pulmonal disease (e.g., insulin-dependent DM-2, lung embolism) **-**Use of anticoagulants (except acetylsalicylic acid monotherapy) **-**Allergy for tissue glue of Healthdot or white bandages* **-**Medication use for rhythm disorders (e.g., betablockers) **-**ICD and/or pacemaker* **-**Skin disorder, rash, wounds, or scars at the area of costa 12 on the left side*

^*^Exclusion criteria for remote monitoring with the Healthdot

On the day of surgery, patients scheduled for SDD were admitted early in the morning. Vital signs were checked, and a blood sample was drawn to get a baseline hemoglobin level. Patients fasted before surgery, with their last meal a minimum of 6 h before surgery. However, patients were allowed to drink a small amount of water in the morning. Upon arrival at the pre-anesthetic room, the anesthesiology protocol was initiated with administration of 1000 mg of paracetamol and antibiotics (cefazolin/metronidazole). Intravenous (IV) fluids (plasmalyte) were started with a total volume of 1000 mL being continued during their clinical stay. For induction of general anesthesia, 1–2 mg/kg propofol, 0.6 mg/kg rocuronium, and 0.2–0.5 µg/kg/min of remifentanil were administered. For maintenance of the anesthesia during the procedure, remifentanil was continued at the same dosage, and sevoflurane was administered through the endotracheal tube at an end-tidal concentration of 1.2%. Additionally, 10 mg of dipidolor and 10 mg of ketamine were administered. At the end of the procedure, 100–200 mg bridion was given. As prophylactic anti-emetics, 4 mg of ondansetron and 8 mg of dexamethasone were given intravenously.

The procedure had to be finished before 13:00 and thus included the first three or four procedures of the day to ensure a minimum observation period of 6 h before discharge was achieved. After surgery, patients were admitted at the daycare facility, and at least 6 h postoperatively, the discharge criteria were checked (see Table 2). To check for the discharge criteria, the postoperative hemoglobin was assessed through a blood sample drawn 6 h after the end of the procedure. If the patient met the discharge criteria, the following one-off medication was given at the ward: dalteparin (Pfizer, USA) and etorixocib (Merck, USA). In addition, at discharge, patients were prescribed the following oral medication: standard dosage paracetamol (1000 g, four times daily) for 5 days. As rescue medication, oxycodone and ondansetron were provided. Oxycodone 5 mg could be used up to four times for the first postoperative day while ondansetron 4 mg could be used up to three times. Of note, very few patients used this rescue medication. In addition, the standard medication following bariatric surgery included omeprazole 40 mg which had to be taken during 6 months, and long-term supplementation with Calcichew 1.25gr/800 ie and multi-vitamins (FitforMe).

**Table 2:** Discharge criteria for same-day discharge

Discharge criteria for approval of same-day discharge (evaluated at least 6 h postoperatively)
-Patient has mobilized (start 1 h after arrival)
-Minimum oral intake of fluids (ice water)
-No severe pain or clinically important PONV
-Maximum decrease in hemoglobin level of 1 mmol/L
-Normal vital signs, MEWS ≤ 2
-Placement of Healthdot and connection with the remote monitoring system
-Administration of 5000 IE dalteparin and 60 mg Etoricoxib
-Approval of bariatric surgeon and patient for discharge

### Healthdot

The Healthdot is a remote single-use wearable sensor that continuously measures the heart rate and the respiration rate. The Healthdot is a class IIa medical device, compliant with the European Union Medical Device Regulation, and it has a CE marking (EU MDR 2017/745). Clinical studies demonstrated a high accuracy in measuring heart rate and respiratory rate with respectively 87.5% and 92.3% of the Healthdot data corresponding to the measurements by the golden standard, the bedside patient monitor [15]. The device can be worn for up to 14 days, with data being wirelessly transmitted to a dashboard. The dashboard can be accessed by specialized eNurses and displays averages calculated over 5-min intervals. The eNurses check the dashboard at least two times a day, always in the morning and in the afternoon. On the surgical ward, the Healthdot is placed and checked for connection to the dashboard before discharge.

If the vital signs rise above the pre-defined threshold, the events will be flagged and a warning score will be calculated. The thresholds are set at a heart rate above 110 beats per minute (2 points) and a respiration rate above 20 per minute (1 points) as in accordance with the literature and other trials [14]. If the warning score was higher than 2, this prompted the specialized eNurses to contact the patient by telephone during office hours to ask about any complaints or exercise. If deemed necessary, the patients were reviewed in the hospital. In case of an alarm outside of the office hours, the alarms are not noted by the patients and will only be seen by the eNurses during the next scheduled check. However, patients are instructed to call the hospital if alarm symptoms occur. They are advised to contact the surgery department where the on-call physician will evaluate the complaints and, if necessary, send the patient to the emergency department.

### Data Collection

All patients were prospectively included in the database after which the data were retrospectively collected from the electronic patient files (EPF). The retrieved baseline data included age, gender, height, weight, body mass index (BMI), ASA classification, and obesity-related complications (i.e., hypertension, diabetes mellitus (DM), obstructive sleep apnea syndrome (OSAS), hypercholesterolemia). Perioperative information regarding surgery duration, length of stay at recovery, and overall stay at the hospital were collected.

The primary outcome measure was the success rate of the SDD, defined as meeting the discharge criteria and being discharged on the same operative day. The SDD is assessed with and without readmissions in the first 48 h. The secondary outcomes included readmissions, short-term (< 30 days) complications according to the Clavien-Dindo classification, and Healthdot alarms.

### Statistical Analysis

Statistical analysis was performed using the IBM SPSS Statistics for Windows, version 29.0. Categorical variables were presented as frequencies with percentages. Continuous variables were presented as mean ± standard deviation (SD) for normal distributed variables and median and inter-quartile range (IQR) for a skewed distribution. Differences between subgroups were tested using a student’s *t-test* or a Mann–Whitney *U* test. A *p*-value of *p* < 0.05 was considered statistically significant. Missing data were reported as such.

## Results

### Baseline Characteristics

A total of 813 consecutive primary bariatric procedures were performed between March 2022 and March 2023. Of these procedures, 299 patients were non-eligible for SDD. The main reasons for non-eligibility were a BMI of 50 or higher (*n* = 75, 25.1%), travel distance to the hospital > 30 min (*n* = 51, 17.1%), or use of anticoagulants (*n* = 32, 10.7%). There were 514 patients who were eligible for SDD following the pre-defined selection criteria (Table 1). Of the eligible patients, 260 were scheduled for an operation according to the SDD protocol, while the remaining 254 eligible patients were operated with a planned overnight stay. The preoperative demographical data of each group are summarized in Table 3. The majority was female with 74.9% in the non-eligible group, 82.7% in the eligible group, and 83.1% in the operated SDD group. The median age was comparable between groups and varied between 40 and 48. Before the procedure, the median BMI at screening was comparable between groups with 42.3 vs 40 vs 41.4 kg/m^2^. The obesity-related complications present at screening in the group operated as SDD consisted of hypertension (*n* = 42), DM (*n* = 9), OSAS (*n* = 33), and dyslipidemia (*n* = 22).

**Table 3:** Baseline characteristics

Baseline characteristics	Non-eligible	Eligible not SDD	Operated as SDD
	*N* = 299	*N* = 254	*N* = 260
Age (years)	48 (37–57)	44 (34–53)	40 (31–52)
Gender			
Male	75 (25.1)	44 (17.3)	44 (16.9)
Female	224 (74.9)	210 (82.7)	216 (83.1)
Type of surgery			
RYGB	269 (90)	220 (86.6)	237 (91.2)
Sleeve	30 (10)	34 (13.4)	23 (8.8)
Height (cm)	169 (163–175)	167 (163–174)	168 (163–173)
Weight at screening (kg)	121 (108–139.5)	113 (103–124)	117 (108–128)
BMI at screening (kg/m^2^)	42.3 (38.4–43)	40 (37.5–42.2)	41.4 (39.3–43.8)
ASA classification			
2	59 (19.7)	76 (29.9)	65 (25)
3	240 (80.3)	178 (70.1)	195 (75)
Obesity-related complications at screening			
Hypertension	129 (43.1)	69 (27.2)	42 (16.2)
Diabetes mellitus	57 (19.1)	15 (5.9)	9 (3.7)
OSAS	66 (22.1)	60 (23.6)	33 (12.7)
Dyslipidemia	72 (24.1)	23 (9.1)	22 (8.5)

Data are presented as mean ± SD, median (IQR), or *n* (%)

Eligible but no SDD patients meet the criteria for SDD but were operated with a planned overnight stay; operated as SDD patients were operated on with the intention of SDD

*SDD* same-day discharge, *RYGB* Roux-en-Y gastric bypass, *OSAS* obstructive sleep apnea syndrome

### Success Rate of SDD

Of these 260 patients, 246 patients (94.6%) could be successfully discharged the same day. The reasons for longer admission at the surgical ward were nausea and malaise (*n* = 6), difficulty mobilizing (*n* = 2), rectal blood loss requiring treatment with tranexamic acid (*n* = 1), decrease in hemoglobin level (*n* = 1), divergent vital signs with tachycardia and fever (*n* = 1), urinary retention (*n* = 1), surgery completed after 13:00 h (*n* = 1), and no caregiver present at home (*n* = 1). In the first 48 h postoperatively, 8 out of the 246 discharged patients were readmitted to the hospital (3.2%), resulting in a successful SDD rate without readmission in the first 48 h of 91.5% (Table 4). Patients spent on average 10:47 h in the hospital, counted from admission to the daycare department till discharge.

**Table 4:** Postoperative outcomes

Same-day discharge	246 (94.6)
Without readmission within 48 h	238 (91.9)
Readmissions POD after SDD	
POD 0–2	8 (3.2)
POD 3–30	3 (1.2)
Complications after SDD within 48 h	
CD 1	1 (0.4)
CD 2	7 (2.7)
Short-time complications after SDD discharge > 48 h and < 30 days	
CD 1	1 (0.4)
CD 2	1 (0.4)
CD 3a	2 (0.8)
CD 3b	1 (0.4)

Data are presented as mean ± SD, median (IQR), or *n* (%). Complications according to Clavien-Dindo classification

*POD* postoperative day, *SDD* same-day discharge, *CD* Clavien-Dindo

### Complications and Alarms

A total of 13 complications (5%) occurred in the first 30 days postoperatively of which 8 (3.1%) happened in the first 48 h and 5 (1.9%) in the period between day 3 and day 30 (Table 4). The eight patients readmitted in the first 48 h all had complications classified as Clavien-Dindo (CD) 1 or 2. Six out of the eight were readmitted due to rectal blood loss. Other admission reasons were general malaise (*n* = 1) and a collapse with a decreased hemoglobin level without rectal blood loss (*n* = 1). All patients were treated conservatively and did not require a re-intervention.

In the period, between 48 h and 30 days, there were three more readmissions (1.2%). The complications consisted of one mild complication (CD2) and two severe complications (CD ≥ 3) requiring re-intervention. The complications requiring re-intervention consisted of a perforation requiring endoluminal stent placement (CD3a) at POD 16 and an infected hematoma due to an anastomotic leakage requiring a laparoscopic procedure (CD3b) at POD 12. Moreover, two complications occurred whereby patients presented themselves at the emergency department but did not require an overnight hospital admission. One patient had an omental infarction whereby conservative treatment was chosen (CD1) and one patient suffered from a food impaction requiring gastroscopy (CD3a). An overall complication rate of 1.2% for complications CD ≥ 3 was found in the first 30 days postoperatively.

Regarding the alarms of the Healthdot device, there were 28 patients of the 217 patients with a Healthdot (12.9%) in which an alarm was monitored during the 7-day period. There were two patients that were readmitted whereby an alarm was noted. One patient with rectal blood loss had tachycardia on the first day while the second patient had an alarm on day 1 leading to the readmission with general malaise. The other 26 patients with alarms had reassuring consultations with the eNurse, and no complications occurred. The remaining 11 patients who had a short-term complication did not have any alarms during the week the Healthdot device was used. In three patients (1.4%), the Healthdot was dysfunctional from the start, and no values were registered. For another 11 patients (5.1%), the Healthdot was removed in the days following the first consultation with the eNurse, resulting in at least 1 day of vital signs being monitored. The reasons for patient-initiated removal were due to complaints of itching or detachment of the device. In six patients, the device stopped sending values after a few days without detachment of the device.

When evaluating the specific complications, e.g., rectal blood loss, the Healthdot only showed an alarm on the day of the complication in one of six patients (16.6%) while another patient had an alarm 6 days after the blood loss occurred (16.6%) without any symptoms at the time of the alarm. In the remaining four patients, no alarm was noted even though a complication occurred (66.7%).

## Discussion

The aim of this study was to assess the feasibility of SDD and the value of remote monitoring with the Healthdot device after primary BMS in the first year after introduction in a large teaching hospital. We demonstrated a success rate of 94.6% (246/260) for SDD and 91.5% (238/260) without readmission in the first 48 h. This success rate is slightly higher than the current literature as Kleipool et al. and Nijland et al. both demonstrated a success rate for SDD without readmission in the first 48 h of 88% [10, 16]. A recent systematic review from Vanetta et al. showed a success rate for SDD between 88 and 98%; however, the readmissions within 48 h were not taken into account in this study [7]. The main reason for an overnight stay (i.e., nausea in 6/14 cases) in the present study was similar to the literature as it was mostly due to nausea [16]. We suggest defining the success rate for SDD as discharge on the same day without readmission within 48 h, rather than just discharge on the same day as it provides a more accurate reflection of patient safety. Reducing readmissions indicates that the patient was discharged in a stable condition, which is a more meaningful indicator of a successful outcome.

This study shows a short-term complication rate of 1.2% within the first 30 days postoperatively for the severe complications classified as Clavien-Dindo ≥ 3a. These findings are low compared to the current literature as a major complication rate of 2.7–6.7% after RYGB and 1.7–6.8% after SG is found [17–19]). Our complication rate of 1.2% is also lower than the 1.6% reported in the Dutch Audit of Treatment for Obesity (DATO), suggesting that lower-risk patients are successfully selected [2]. This aligns with the findings of Kleipool et al., who applied similar selection criteria [16]. This emphasizes the importance of selecting low-risk patients for a safe introduction of SDD and is one of our key messages: SDD can safely be implemented with an appropriate patient selection.

The majority of the patients (63.2%) were eligible for SDD based on the clinical criteria. However, due to logistical limitations, not all eligible patients could be scheduled for discharge on the same day. The primary reason was the requirement that patients had to be operated on before 13:00 to be discharged the same day as this allowed sufficient time for postoperative monitoring. Not all surgeries could be scheduled and completed before this time, resulting in many eligible patients having to remain in the hospital overnight.

The present study uses the Healthdot for remote monitoring whereby an alarm occurred in 12.9% of the patients of which only two patients (0.9%) were readmitted following the alarm. The literature regarding remote monitoring in bariatric surgery is limited. The study of Nijland et al. uses remote monitoring during 48 h and demonstrated that no abnormal vital signs occurred in relation to complications [10]. However, in their study, the monitoring was not continuous but rather consisted of video consultations and measurements performed by patients themselves, which can influence the detection of real-time complications. Currently, the PEACH trial is being conducted to assess telemonitoring with the Healthdot in SDD which is the same device used in our study which would make a more interesting comparison [14]. One of the hypotheses as to why monitoring does not adequately predict complications might be that the current monitoring systems often rely on pre-defined cutoff values for vital signs such as heart rate or respiratory rate. This approach may miss subtle changes or trends that occur before a complication fully develops. A more effective approach could involve evaluating the monitor trends over time by detecting the small, cumulative changes in vital signs. This may allow for earlier identification of complications.

While remote monitoring may not predict complications, it could still provide other benefits, such as enhancing patient satisfaction and ensuring a feeling of safety. Though not extensively analyzed in this study, it is thought that patients may feel more secure due to the continuous monitoring. Especially during the implementation phase of SDD, remote monitoring could serve as a tool to facilitate the transition from overnight to SDD, as was the case in this teaching hospital. However, once the SDD protocol becomes standard care, it remains uncertain whether continued monitoring is still necessary or beneficial.

### Limitations

This study has some limitations that need to be acknowledged. First, no randomization between SDD and overnight stay was performed which may introduce selection bias. The possible selection bias itself is also a limitation. The decision for SDD is based on clinical judgement or patient factors, and thus, the group might differ in ways that could influence outcomes. However, based on the baseline characteristics, no significant differences are found between these groups suggesting that the potential bias may be minimal.

### Further Research

In our study, the addition of remote monitoring was not proven beneficial. However, for future research, the value of monitoring needs to be examined in other groups such as patients undergoing revisional surgery or in more complex patients with a higher risk of complications. In addition, further research is needed to assess whether dynamic monitoring, which analyzes patterns and trends over time, can serve as a more effective method for predicting complications.

## Conclusion

Based on this study, the first year of same-day discharge following primary bariatric metabolic surgery in a large teaching hospital had a high success rate of more than 90% following a specific selection. The demonstrated complication rate is lower than the literature for patients with an overnight stay, supporting the fact that low-risk patients were selected. Secondly, the Healthdot does not adequately predict the occurrence of complications that require either admission or re-intervention when using the current alarm settings. Implementation in more high-risk BMS patients might be of interest.

## Data Availability

No datasets were generated or analysed during the current study.
